# Fair Facial Attribute Classification via Causal Graph-Based Attribute Translation

**DOI:** 10.3390/s22145271

**Published:** 2022-07-14

**Authors:** Sunghun Kang, Gwangsu Kim, Chang D. Yoo

**Affiliations:** School of Electrical Engineering, Korea Advanced Institute of Science and Technology, Daejeon 34141, Korea; sunghun.kang@kaist.ac.kr (S.K.); s88012@kaist.ac.kr (G.K.)

**Keywords:** deep learning, fair classification, facial attribute classification

## Abstract

Recent studies have raised concerns regarding racial and gender disparity in facial attribute classification performance. As these attributes are directly and indirectly correlated with the sensitive attribute in a complex manner, simple disparate treatment is ineffective in reducing performance disparity. This paper focuses on achieving counterfactual fairness for facial attribute classification. Each labeled input image is used to generate two synthetic replicas: one under factual assumptions about the sensitive attribute and one under counterfactual. The proposed causal graph-based attribute translation generates realistic counterfactual images that consider the complicated causal relationship among the attributes with an encoder–decoder framework. A causal graph represents complex relationships among the attributes and is used to sample factual and counterfactual facial attributes of the given face image. The encoder–decoder architecture translates the given facial image to have sampled factual or counterfactual attributes while preserving its identity. The attribute classifier is trained for fair prediction with counterfactual regularization between factual and corresponding counterfactual translated images. Extensive experimental results on the CelebA dataset demonstrate the effectiveness and interpretability of the proposed learning method for classifying multiple face attributes.

## 1. Introduction

Facial attribute classification detects the presence versus absence of various labeled attributes, including bio-metric features (“big nose”), expression (“smiling”), and worn accessories (“glasses”). Attribute classification supports various tasks including tagging, searching, detecting, and verification of identity [1,2,3,4,5,6,7,8]. Existing facial attribute classification algorithms have solely been focused on prediction accuracy and are highly likely to suffer from prediction bias that leads to disparity in performance among various population subgroups belonging to different gender, race and age. In fact, recent studies have exposed the racial and gender disparity in the performance of a number of commercial face recognition systems. For public and commercial use, prediction must be fair.

Although considerable research in AI has been devoted to mitigating general prediction bias for fair prediction, a substantial portion of the research had been centered on attaining group fairness [9,10,11,12,13] which is focused on reducing performance disparity among groups with distinct sensitive attributes. This notion of group fairness does not necessarily equate well with the interest of an individual [12,14]. For any system that affects the welfare or opportunities available to an individual, individual fairness should be considered over group fairness. The counterfactual fairness is an attractive alternative as it captures the intuition that a decision is fair towards an individual if it is the same in (a) the actual world and (b) a counterfactual world where the individual belonged to a different demographic group. It requires the probability distribution for the individual’s classifier output label to be unchanged regardless of the switched value of the sensitive attribute [15].

With the aid of deep learning [16,17,18,19], face recognition performance has substantially improved to the point of exhibiting close to human performance. However, a number of studies [20,21] have noted disparity in prediction performance among groups with different sensitive attributes that include gender and race. It is observed that prediction accuracy is lower for African-Americans and females than for Caucasians and males. In an attempt to resolve this issue, a method based on unsupervised domain adaptation IMAN [20] has been proposed to improve recognition performance for ethnic minority groups.

One common cause for prediction bias comes as a consequence of a trained model inheriting the bias in the training dataset [22]: the sampling bias and societal bias [23,24] in the dataset lead to skewed distributions with respect to the sensitive attribute. One measure to counteract this bias inheritance is to collect a more balanced dataset. FairFace [25] with an emphasis on balanced mixed-race composition was released for developing classification models whose accuracy is consistent between race and gender groups. However, collecting a dataset with balanced facial attributes is a challenging task for a number of reasons including (1) societal bias, e.g., younger people, more than seniors, take photos and upload them on the web, and (2) the complex relationship that exists among facial attributes, leading to a greater-than-exponential rise in the number of training images that must be collected in order to obtain constant minimum sample sizes for all combinations of a linearly increasing number of attributes. These factors make fair data collection nearly impossible.

To achieve fair face-attribute classification, an algorithm is required that mitigates bias regardless of the sample imbalance and the existence of complex relationships among attributes. This paper studies a learning method that focuses on achieving counterfactual fairness for face-attribute classification based on an encoder–decoder framework that translates the input image into both factual and counterfactual images using a causal graph of the attributes. More specifically, from the facial attribute labels in the training dataset, the complex relationships between the facial attributes and the sensitive attribute are modeled and represented by a causal graph. Observed input facial attributes seed a sampling algorithm that generates the attribute lists for faces with factual and counterfactual settings of the sensitive attribute, based on the discovered causal graph. The causal graph-based attribute translator generates realistic factual and counterfactual images corresponding to the observed image and sampled counterfactual facial attributes. The attributes of the generated counterfactual image follow the intervened attribute distribution represented by the causal graph. Based on the factual and counterfactual image and label pair, a counterfactual regularization term is proposed that penalizes differences between the prediction probabilities of the counterfactual and factual images, thereby encouraging the learning of a counterfactually fair face attribute classifier. The counterfactual regularization penalizes prediction differences only for attributes with the same sampled value, thereby learning fairness without the need to build a separate causal graph for each attribute.

Our contributions can be summarized as follows:

- An encoder–decoder framework is proposed, incorporating a causal graph of face attributes, to facilitate input image translation in generating factual and counterfactual images for counterfactually fair learning.
- A counterfactual regularization term is incorporated to penalize counterfactually unfair facial attribute classification, thereby reducing the counterfactual fairness disparity in multi-label classification.
- We demonstrate the overall framework can achieve counterfactual fairness on the CelebA corpus, and we also provide factual and counterfactual images with multi-dimensional attributes induced from the causal graph.
- This work, to the best of our knowledge, is the first attempt in providing counterfactual fairness considering the complicated causal relationships among face attributes, including sensitive attributes, for fair facial-attribute classification.

The remainder of this paper is organized as follows. Section 2 briefly reviews some of the most relevant literature related to the proposed framework. Section 3 describes counterfactual fairness for face attribute classification. Section 4 describes the proposed learning method to achieve counterfactual fairness for face attribute classification. Experimental results are reported in Section 5. Section 6 provides a summary and conclusion of the paper.

## 2. Related Work

### 2.1. Fairness in Computer Vision

A number of studies [21,26] have raised concerns regarding unfair prediction in commercialized recognition systems, and a number of studies [20,27,28,29,30,31,32] have been conducted to achieve fair prediction on various computer vision tasks. Several image captioning and VQA models [27,33,34] have been noted to exaggerate biases [27,34] that lead to incorrect captions due to over-reliance on prejudicial context. To mitigate bias, the models focus on the object, not the context. In the image classification task, the Blind network [29] introduces confusion loss, which penalizes non-uniformly distributed predictions of the sensitive attribute in order to learn fair representation. Domain adaptation is one of the methods to reduce the performance gap between different ethnic groups [20]. The information of the major groups can be transferred to minor groups for reducing the performance gap.

### 2.2. Counterfactual Fairness

In contrast to other group fairness measures, the counterfactual fairness measure [35,36,37] is grounded on the causal relationship among variables. The counterfactual fairness criterion states that a prediction is fair toward an individual if the predictions of the actual world and counterfactual world are the same regardless of the sensitive attribute. The counterfactual is obtained by intervention in the causal graph. The origin of biased prediction can be analyzed using the causal graph represented as a Bayesian network [38]. It provides a graphical interpretation of unfairness in a dataset as the presence of an unfair causal path in the causal Bayesian network. Chiappa and Issac [39] analyze the fair decision system in complex scenarios where a sensitive attribute might affect the decision along both fair and unfair pathways in a causal model.

For computer vision tasks, Gender Slopes [40] are proposed for evaluating counterfactual fairness. Gender Slopes synthesize counterfactual images using an encoder–decoder framework, then measure the counterfactual fairness gap. The study of [40] reveals that state-of-the-art commercial computer vision classifiers produce biased predictions against gender attributes. However, the Gender Slopes do not consider the causal relationship between variables including the sensitive attribute. In facial attribute classification, Denton et al. [41] proposed measuring counterfactual fairness by generating a facial image with a Generative Adversarial Network (GAN). Here, the effect of an orthogonal vector to the decision boundary of each attribute classifier on a one-dimensional smiling classifier is investigated.

### 2.3. Image Generation

Goodfellow et al. [42] proposed a framework for training a deep generative model in an adversarial manner referred to as the generative adversarial network (GAN). This framework simultaneously trains two different networks to mimic the data distribution: (1) a discriminator is designed to distinguish between the generated and original sample, and (2) a generator is trained to generate samples realistic enough to fool the discriminator. Building on the success of the GAN, various studies have been conducted to generate more realistic images. PGGAN [43] is proposed to increase the image resolution progressively during the training. Recently, BigGAN [44] is proposed to generate more realistic images with deeper architecture. Causal GAN [45] is proposed to sample both the observational and intervention distribution. They use an additional GAN for controlling the causal relationship.

There are also some conditional GANs [46,47] to generate new images from given images. AttGAN [48] is one of the most successful architectures for generating facial images with discretely modified facial attributes. The additional classifier, which predicts the facial attributes of generated images, provides supervision indicating which attributes should appear in the generated facial images.

This paper differs from CausalGAN and AttGAN in that: the proposed causal graph-based attribute translator generates the counterfactual images from a given image and facial attributes. The CausalGAN [45] is proposed to generate facial images considering the causal relationship among the attributes; the CausalGAN can sample facial images from the intervened distribution of the causal relationship discovered from the training facial image and attribute vector pairs. However, CausalGAN does not guarantee the generated facial images keep the identity of the given images, and it cannot generate counterfactual images. To generate counterfactual face images of a given observed sample, a causal graph that explains the data distribution is required. The conditional GAN algorithms [44,46,47,48], including AttGAN, require the desired attribute to generate the given facial image without consideration of the causal relationship. Without a causal graph that describes the relationship between the attributes and the image, the conditional generation algorithm cannot generate counterfactual facial images.

## 3. Problem Definition

Given facial image $x\in X\subset R^{c\times w\times h}$, where c,w, and *h* are the dimensions of channel, width, and height, respectively, our task is to predict binary ground truth label $y=(y_{1},y_{2},\ldots ,y_{M})$ where $y_{i}\in \left\{0,1\right\}$ for i=1,…,M representing the presence of each of the *M* attributes. Here, the elements of y are correlated. A classifier parameterized by θ, $f_{\theta }$, is learned to predict the presence of *M* facial attributes as $\hat{y}=I\left(\hat{p}>0.5\right)$, where $\hat{p}=f_{\theta }\left(x\right)$ and *I* is an indicator function. Here, $f_{\theta }$ is assumed to be a convolutional neural network (CNN), and θ is learned to minimize the summation of cross-entropy losses between the ground-truth attribute vector $y_{i}$ and ${\hat{y}}_{i},i=1,\ldots ,M.$

For the counterfactual images, we assume a causal graph satisfying the SCM (Structured Causal Model) condition in [15] and modeling the relation between image*x*, attributes *Y* and sensitive attribute A. Then, we conduct (1) abduction, (2) action, (3) prediction. The $X_{A\leftarrow a^{\prime }}$ denotes the image generated by the intervention.

Then we define the counterfactually fair classifier $f_{\theta }{\left(\cdot \right)}_{i}$ for the *i*-th attribute of y as:

(1) $$\begin{array}{c} P(f_{\theta }{\left(X_{A\leftarrow a}\right)}_{i}=y_{i}\left|X=x,A=a\right)\\ \;=P(f_{\theta }{\left(X_{A\leftarrow a^{\prime }}\right)}_{i}=y_{i}|X=x,A=a). \end{array}$$

Spontaneously the counterfactual fairness is measured by the counterfactual disparity (CDP) as follow:

(2) $$\begin{array}{c} \begin{array}{cc} CDP_{i}\left(x,a\right)= & |P\left(f_{\theta }{\left(X_{A\leftarrow a}\right)}_{i}=y_{i}|X=x,A=a\right)\\ & -P\left(f_{\theta }{\left(X_{A\leftarrow a^{\prime }}\right)}_{i}=y_{i}|X=x,A=a\right)|, \end{array} \end{array}$$

where i=1,…,M. The goal of this work is to simultaneously minimize the attribute prediction error $err(\hat{y},y)$ and the counterfactual disparity $CDP_{i}\left(x,a\right)$ for each given face image.

## 4. Method

The proposed learning method consists of the following three procedures: (1) Discovering the causal relationships among the attributes to be used in a detailed causal graph, (2) learning the causal graph-based attribute translator for generating counterfactual images $X_{A\leftarrow a^{\prime }}$ from the given image, sensitive attribute, and causal relationship, and (3) learning the counterfactually fair facial attribute classifier with counterfactual regularization.

**Figure 1:**
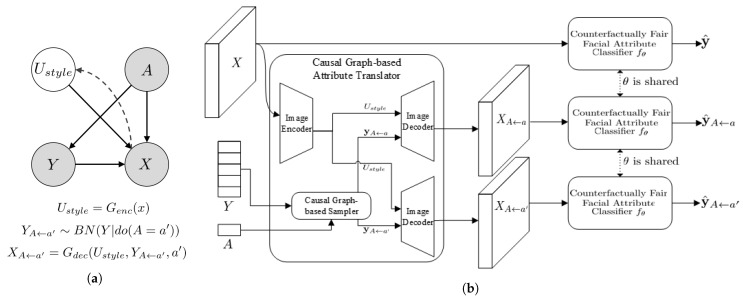
Illustration of the proposed framework. (**a**) The generation and inference process of the causal graph-based counterfactual attribute translator. Solid and dashed lines indicate the generation and inference process, respectively. The relationships between the sensitive attribute *A* and facial attributes *Y* are modeled in Bayesian Network (BN). The encoder $G_{enc}$ and $G_{dec}$ are modeled in autoencoder with adversarial loss. (**b**) The proposed learning framework. From the observed image *X*, two different types of counterfactual images are generated using the Causal Graph-based Counterfactual Attribute Translator. The total loss function consists of cross-entropy loss between $\hat{y}$ and y and the counterfactual regularization that absolute difference between predictions of the generated facial images ${\hat{y}}_{A\leftarrow a}$, and ${\hat{y}}_{A\leftarrow a^{\prime }}$. where $Y^{\prime }$ is generated on the intervention of $A=a^{\prime }.$ $X_{A\leftarrow a^{\prime }}=G_{dec}\left(U,y^{\prime },a^{\prime }\right)$ where $y^{\prime }$ is a sample from intervened distribution of *Y*.

The overall framework for the proposed learning method is illustrated in Figure 1. Details of the figure are discussed below.

### 4.1. Discovering the Causal Relationship of Attributes

A face image is encoded into the following three variables: *A*, *Y*, and $U_{style}$. Here, $U_{style}$ is a latent variable independent of *Y* and *A* [48]. To generate counterfactual face images, it is necessary to discover the causal relationship among *Y* and *A*.

In this paper, causal discovery based on a data-driven method is used to avoid additional biases from human perception. The causal relationships among *Y* and *A* are discovered by Graphical Lasso [49], and Greedy Equivalence Search (GES) [50] algorithms. The search space (the number of potential causal relationships among attributes) grows exponentially as the number of facial attributes increases. The Graphical Lasso provides sparse un-directed relationships between attributes, which helps to reduce the search space of causality discovery algorithms such as GES. The learned structure of the causal model is represented as an Adjacency matrix ($A_{adj}$).

Using the discovered causal graph structure, a Bayesian Network is trained to estimate the weights of causal relationships among attributes *Y* and *A*. Then, we can have the causal model to address the dependency between attributes, which can be used for the prediction of counterfactual images.

### 4.2. Causal Graph-Based Attribute Translator for Counterfactual Images

The causal graph-based attribute translator is designed to generate multiple counterfactual facial images corresponding to the observed face image. It consists of two submodules: Encode for the *abduction* for $U_{style}$ in the proposed causal model, (2) attribute translator for the *action*, (3) decoder for the *prediction.* To do this, we use the convolutional neural network G, composed of the encoder $G_{enc}$ and decoder $G_{dec}$ connected to the casual graph-based sampler for attributes. The encoder $G_{enc}$ is for the *abduction*, and the decoder $G_{dec}$ combined with causal graph sampler is for the *action* and *prediction*. Figure 1a represents the generation and inference process of the Causal Graph-based Attribute Translator.

The image encoder $G_{enc}$ for the it abduction takes the observed image x,y, and *A*. and extracts the latent representation $U_{style}$, This is given below:

$$U_{style}=G_{enc}\left(x,y,A\right),$$

which is to model the posterior of $U_{style}.$

In the attribute translator for the *action*, the counterfactual attribute vectors are sampled from the intervened attributes distribution. In sampling, we use the likelihood-weighted particle generation [51].

Then the image *X* is predicted by the decoder $G_{dec}$ with the sampled attributes. The image decoder $G_{dec}$ generates a realistic face image from the latent representation $U_{style}$ and the sampled counterfactual attribute vector $y_{A\leftarrow a^{\prime }}.$ This is given below:

$$x_{A\leftarrow a^{\prime }}=G_{dec}\left(U_{style},y_{A\leftarrow a^{\prime }},a^{\prime }\right).$$

To sum up, the neural network G, referred to as casual graph-based attribute translator, generates a counterfactual image with respect to a given observed image *x* and counterfactual attribute $y_{A\leftarrow a^{\prime }}$:

(3) $$\begin{array}{c} x_{A\leftarrow a^{\prime }}=G\left(x,y_{A\leftarrow a^{\prime }},a^{\prime }\right), \end{array}$$

and we consider two types of intervention distribution [52], i.e., $X_{A\leftarrow a}$ and $X_{A\leftarrow a^{\prime }}$ because our aim is to construct the counterfactually fair classifier.

What remains is how to train the causal graph-based attribute translator. Two auxiliary classifiers are created to train the *G*: (1) A discriminator *D* provides the supervision for generating realistic images, and (2) an auxiliary attribute classifier *h* forces the intervention image to have the sampled counterfactual attribute vector. The causal graph-based attribute translator is obtained from the training using the following losses:

$$\begin{array}{ccc} L_{D,adv} & = & -E_{\left(x,y\right)}\left[D\left(x\right)\right]+E_{\left(x,\tilde{y}\right)}\left[D\left(G\left(x,\tilde{y},\tilde{a}\right)\right)\right]],\\ L_{G,adv} & = & -E_{\left(x,\tilde{y}\right)}\left[D\left(G\left(x,\tilde{y},\tilde{a}\right)\right)\right]],\\ L_{h,cls} & = & E_{\left(x,y\right)}\left[CE\left(h\left(x\right),y\right)\right],\\ L_{G,cls} & = & E_{\left(x,\tilde{y}\right)}[CE\left(h\left(G\left(x,\tilde{y},\tilde{a}\right),\tilde{y}\right)\right],\\ L_{l1} & = & {∥G\left(x,y,a\right)-x∥}_{1}, \end{array}$$

where CE is cross-entropy loss and $\tilde{y}$ follows the mixture distribution of $0.5Y_{A\leftarrow a}+0.5Y_{A\leftarrow a^{\prime }}$. Here, the $\tilde{a}$ is tied with the $\tilde{y}$ that if the $\tilde{y}$ is sampled from $Y_{A\leftarrow a}$ then the value of $\tilde{a}$ is *a*, otherwise $a^{\prime }$. The optimization for G,D, and *h* are formulated as below, with hyperparameters $\lambda_{G,cls},\lambda_{h,cls},\lambda_{l1}$:

$$\begin{array}{ccc} G^{*} & = & \arg\min_{G}\left\{L_{G,adv}+\lambda_{l1}L_{l1}+\lambda_{G,cls}L_{G,cls}\right\},\\ D^{*},h^{*} & = & \arg\min_{h,∥D∥\leq 1}\left\{L_{D,adv}+\lambda_{h,cls}L_{h,cls}\right\}. \end{array}$$

The neural networks G,D, and *h* are optimized, and the causal graph-based attribute translator is obtained by the optimum $G^{*}.$

### 4.3. Counterfactually Fair Classifier

The causal graph-based attribute translator provides the generated counterfactual facial images corresponding to the given facial image. We propose the counterfactual regularization loss, shown below, for reducing the counterfactual disparity between predictions of factual and counterfactual images:

$$\begin{array}{cc} L_{cf\_reg}\left(x,a\right)=\frac{1}{M}\sum_{i=1}^{M} & |P\left({\hat{y}}_{A\leftarrow a,i}=0|X=x,A=a\right)-P\left({\hat{y}}_{A\leftarrow a^{\prime },i}=0|X=x,A=a\right)|, \end{array}$$

where ${\hat{y}}_{A\leftarrow a,i}=f_{\theta }{\left(X_{A\leftarrow a}\right)}_{i}$ and ${\hat{y}}_{A\leftarrow a^{\prime },i}=f_{\theta }{\left(X_{A\leftarrow a^{\prime }}\right)}_{i}$. Note that $\sum_{y_{i}=0}^{1}P\left({\hat{y}}_{A\leftarrow a,i}=y_{i}|X=x,A=a\right)=1.$ The facial attribute classifier $f_{\theta }$ is a deterministic function of the input facial image, implemented as a convolutional neural network. And $X_{A\leftarrow a^{\prime }}$ is random based on the causal-graph. To estimate $P({\hat{y}}_{A\leftarrow a,i}=0|X=x,A=a)$, we take the strategy of sampling multiple images from two intervention distributions $X_{A\leftarrow a}$ and $X_{A\leftarrow a^{\prime }}$ based on the causal graph. Note that

$$x_{A\leftarrow a}=G^{*}\left(x,y_{A\leftarrow a},a\right),\;y_{A\leftarrow a}∼\mathrm{BN}\left(Y|do\left(A=a\right)\right),$$

$$x_{A\leftarrow a^{\prime }}=G^{*}\left(x,y_{A\leftarrow a^{\prime }},a^{\prime }\right),\;y_{A\leftarrow a^{\prime }}∼\mathrm{BN}\left(Y|do\left(A=a^{\prime }\right)\right).$$

The classification loss for facial attributes is as follow:

$$L_{cls}\left({\hat{p}}_{i},y_{i}\right)=y_{i}\cdot \log\left({\hat{p}}_{i}\right)$$

where $y_{i},{\hat{p}}_{i}$ denote the *i*-the attribute of ground truth attribute vector y and its predicted probability, respectively. The counterfactual regularization $L_{cf\_reg}$ is added to the classification loss $L_{cls}$. The overall loss function ($L_{cf}$) for the counterfactually fair classifier $f_{\theta }$ is follows:

$$L_{cf}\left(x,y,a\right)=\frac{1}{M}\sum_{i=1}^{M}L_{cls}\left({\hat{p}}_{i},y_{i}\right)+\lambda_{cf}L_{cf\_reg}\left(x,a\right).$$

During the training, we calculate the regularization loss of only those attributes whose intervention predictions ${\hat{y}}_{A\leftarrow a},{\hat{y}}_{A\leftarrow a^{\prime }}$ (sampled from both intervention attribute distributions, do(A=a) and $do(A=a^{\prime })$) are the same.

## 5. Experiments

### 5.1. Dataset

Large-scale CelebFace Attributes (CelebA) [2] is used for evaluating the proposed learning method for counterfactual fairness. The dataset consists of approximately 200,000 cropped and aligned facial images. Each facial image has 40 manually labeled binary attributes. In this work, the 29 attributes are selected to reduce the effect caused by extremely imbalanced label distribution; some attributes have too few observed samples in the training dataset to learn a causal controller. Gender attribute is selected as the sensitive attribute, and we conduct experiments around mitigating the bias caused by the different gender in each individual. Attributes are discarded when either the number of attributes samples is less than two samples for reliably predicting the attribute or there is an extreme imbalance in that the number of samples exceeds more than 100 times between the two demographic groups (male and female). The training, validating, and testing partition for experiments is the same as for the pre-defined set.

### 5.2. Experimental Details

For all experiments in this paper, the *gender* and the other 29 facial attributes were selected for the sensitive *A* and target attribute vector y, respectively.

The causal graph-based attribute translator is trained to generate facial images with (128×128) width and height resolution. In this work, the architecture of the proposed attribute translator follows AttGAN [48] design, one of the widely used architectures for generating facial images with given attributes. To train the proposed attribute translator, Adam optimizer [53] is used with a $2\times 10^{-4}$ learning rate. The gradient penalty [54] is adopted for stable training of adversarial loss. The values of $\lambda_{G,cls},\lambda_{h,cls},\lambda_{l1}$ are set to 100.0,10.0, and 1.0, respectively along the AttGAN [48] to match the scale of different types of losses. The number of samples in the intervention distribution is set to 100 for each image observed for training, validation, and testing. The pre-trained ResNet-18 [55], one of the widely used convolutional neural networks in various computer vision tasks, is used as the backbone of the face attribute classifier. The framework is not limited to specific architecture design, which means the causal graph-based attribute translator and the counterfactually fair classifier could be replaced with a state-of-the-art conditional generator and facial attribute classifier.

Counterfactual augmentation is an algorithm that can be compared based on a common causal graph. Counterfactual augmentation is a method to address factual and counterfactual face images during the training of the face attribute classifier. During the training of the facial attribute classifier of both counterfactual augmentation and the proposed learning method, the causal graph-based facial attribute translator is used to generate factual and counterfactual facial image generation. In addition, “baseline” is an experiment evaluated using the counterfactual image generated by counterfactual augmentation of facial image translation to compare the counterfactual fairness performance of counterfactual augmentation and the proposed learning.

The classifier is trained using the union set of observed images and generated images using cross-entropy loss without counterfactual regularization. In the experimental results, the counterfactual augmentation is referred to as cf_aug. The proposed counterfactually fair classifier is denoted by “cf_reg” in the experimental results. The hyperparameter for counterfactual regularization loss, $\lambda_{cf}$, controls the effectiveness of the counterfactual regularizer. This parameter provides the controllability of the trade-off relationship between counterfactual parities and facial attribute classification accuracy. The range of $\lambda_{cf}$ in Section 5.5 is determined to examine the trade-off relationship between accuracy and counterfactual parities. All the experiments of this paper were conducted on a single Titan V, which has 12 GB of memory.

### 5.3. Causal Graph-Based Attribute Translator

#### 5.3.1. Discovered Causal Structure

**Figure 2:**
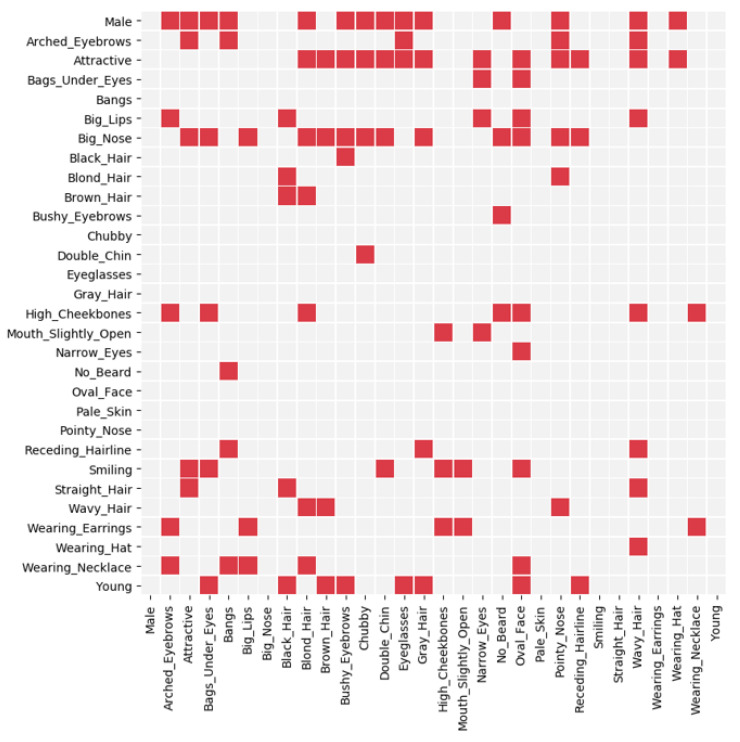
The discovered causal structure among the y and *A*. The red and gray rectangles denote the existence and absence of cause and effect, respectively.

The discovered adjacency matrix representing causal structure is illustrated in Figure 2. The *y*-axis and *x*-axis indicate the cause and the effect, respectively. According to the discovered adjacency matrix, the gender attribute affects many other attributes directly or indirectly. The attributes which are influenced by gender are listed in Table 1.

In total, 14 of 29 attributes are directly affected, and 6 of the remaining attributes are indirectly affected. The number of edges is 104 which indicates that there are complex causal relationships among the facial attributes y and the sensitive attribute *A*. For example, the attribute *Eyeglasses* is influenced by gender in the discovered causal structure, possibly because female celebrities tend to wear contact lenses instead of eyeglasses.

#### 5.3.2. Sampled Counterfactual Attributes

**Figure 3:**
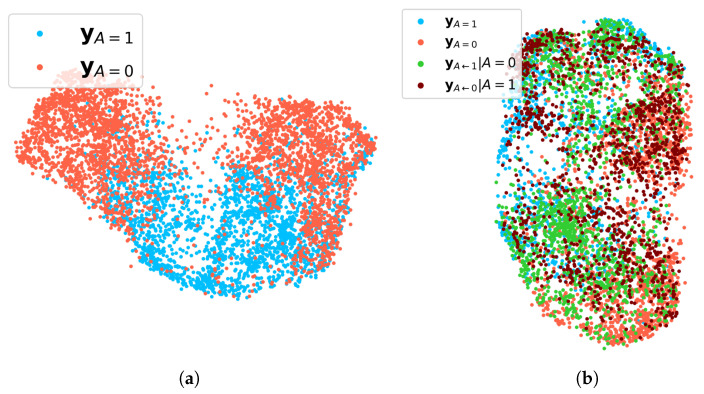
The UMAP visualization is applied for 29 binary facial attributes y without the sensitive attribute (gender). (**a**) Visualization for observed y in the training dataset. Facial attribute vectors of female ($y_{A}=0$) and male ($y_{A}=1$) are colored in orange and blue, respectively. (**b**) Visualization for observed y, and intervened $y_{A\leftarrow a^{\prime }}$. The counterfactual attribute vectors of female and male are colored in green and dark red, respectively.

One of the major motivations of this work is that the sensitive attribute has indirect influences on the facial attributes y. To visualize the concealed influence of gender, UMAP [56] is used. In Figure 3a, y of each demographic group which shares the same sensitive attribute is easily distinguished from the other demographic group. On the other hand, when both y and $y_{A\leftarrow a^{\prime }}$ are represented in UMAP at the same time, the demographic groups cannot be distinguished in Figure 3b. Based on the UMAP visualizations, the counterfactual attribute vector $y_{A\leftarrow a^{\prime }}$ helps to narrow down the gap between the demographic groups.

#### 5.3.3. Generated Counterfactual Images

**Figure 4:**
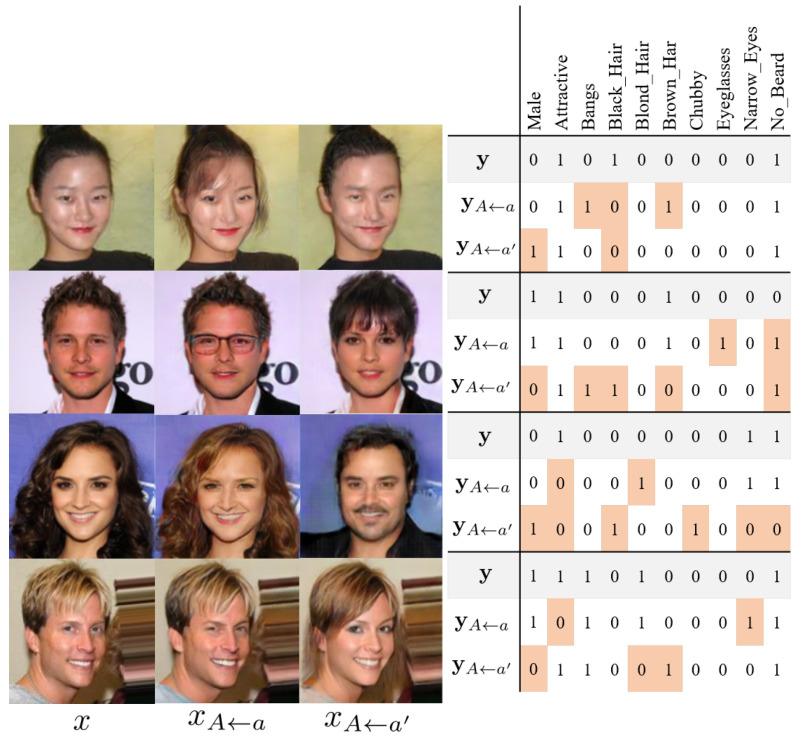
Observed and sampled images from intervention distribution on the gender. Nine of the twenty-nine target attributes and the sensitive attribute are visualized for y, $y_{A\leftarrow a}$, and $y_{A\leftarrow a^{\prime }}$. The gray and red cells indicate observed facial attributes and their values switched due to the interventions, respectively.

The generated images with the intervention attribute distribution which corresponds to the observed image *x* are illustrated in Figure 4.

The pairs labeled (x,y), $\left(x_{A\leftarrow a},y_{A\leftarrow a}\right)$, and $\left(x_{A\leftarrow a^{\prime }},y_{A\leftarrow a^{\prime }}\right)$ are the pairs observed in the test dataset, pairs sampled from an intervention distribution with the same value of the sensitive attribute, and pairs sampled from a counterfactual distribution, respectively. The proposed causal-graph based attribute translator can generate realistic facial images from the intervention attribute and given image. In Figure 4, the attributes of translated images are matched to the intervention attribute vectors.

### 5.4. The Effects of the Counterfactual Regularization

The expectation of the counterfactual disparity over the face images in test partition $E\left(CDP_{i}\right)=E_{x,a}\left[CDP_{i}\left(x,a\right)\right]$ is used to measure counterfactual fairness for each attribute to focus the statistics of prediction over the images in test partition. The averaged counterfactual disparity of the baseline, cf_aug and proposed counterfactual regularization with different balancing hyperparameter $\lambda_{cf}$ are compared in Table 2.

The averaged counterfactual disparities for the baseline facial attribute classifier is measured to be approximately 0.082. The largest value of averaged counterfactual disparity is 0.202 for *Arched_Eyebrows* attributes. With the proposed counterfactual regularization, the trained counterfactually fair facial attribute classifier achieved 0.015 as the averaged counterfactual disparity for *Arched_Eyebrows* attribute. The averaged counterfactual disparities for over 27 attributes except for 2, reduces with the proposed counterfactual regularization. The lowest value of averaged counterfactual disparity is achieved with the cf_reg at $\lambda_{cf}=0.1$. With the larger value of $\lambda_{cf}$, the measured averaged counterfactual parities are decreased. Taking advantage of the fact that the counterfactual disparity $CDP_{i}\left(x,a\right)$ can be measured from each individual image, we compare the variance of $CDP_{i}\left(x,a\right)$. The meaning of the $Var(CDP_{i}\left(x,a\right))$ is the statistics for the degree of inconsistency in predicting *i*-th attribute by each individual. The averaged $Var(CDP_{i}\left(x,a\right))$ values over all attributes are 0.015, 0.014, and 0.004 for baseline, cf_aug, and cf_reg, respectively. Especially, the values of variances for attributes *Eyeglasses, Smiling, Attractive* are reduced remarkably, relative to $E(CDP_{i})$. Note that the inference speed of the counterfactually fair classifier is the same as ResNet-18 [55] and there is no overhead during the inference because the attribute translator is used during the training procedure but not the inference procedure.

### 5.5. Trade-Off Relationship

**Figure 5:**
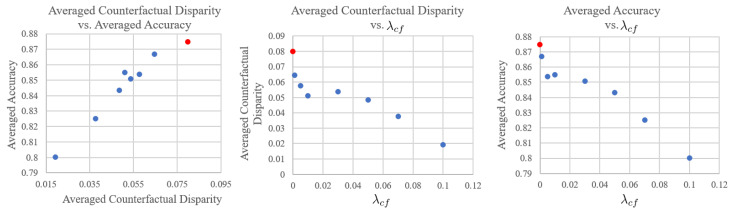
Trade-off analysis of the proposed learning method for counterfactual fairness. Here, the averaged counterfactual disparity is defined as the aggregation over all $E(CDP_{i})$ and the averaged prediction accuracy in the same manner. Each blue dot is obtained with a different value of $\lambda_{cf}$, and the red dot represents baseline performance.

The trade-off relationships between (1) averaged counterfactual fairness vs. averaged accuracy, (2) averaged counterfactual disparity vs. $\lambda_{cf}$, and (3) averaged accuracy vs. $\lambda_{cf}$ are plotted in Figure 5. The larger value of $\lambda_{cf}$ tends to possess less averaged counterfactual disparity, meaning that it is more fair, but the averaged accuracy is reduced. The counterfactual disparity and prediction accuracy have a linear relationship, and the decrease relative to the baseline (red dots in Figure 5) is sharper for the counterfactual fairness.

## 6. Conclusions and Discussion

This paper focuses on achieving counterfactual fairness for face attribute classification using a causal graph-based attribute translation. The proposed causal graph-based attribute translator consists of two submodules: (1) the Bayesian Network for modeling intervention attribute distribution and (2) an encoder–decoder framework for generating the intervention images from the given observed image. The counterfactual regularization, which reduces the counterfactual disparities, is proposed for multi-label classification. In the experimental results on the CelebA dataset, the intrinsic influences caused by the sensitive attribute are discussed, and the proposed causal graph-based attribute translator generates realistic intervened images from the observed face image through sampled intervened attribute vectors on the sensitive attribute. The comparison results show that the proposed learning method reduces both mean and variance of counterfactual disparity with a huge gap. The trade-off relationship between the accuracy and the counterfactual disparity with different hyperparameters is explored. Our work addresses fair facial attribute classification, and can be extended to various tasks such as identity verification and tagging systems.

## Figures and Tables

**Table 1:** Influenced attributes by *gender* in the CelebA.

Sensitive Attribute	Male
Directly Influenced Attributes	Arched_Eyebrows, Attractive, Bags_Under_Eyes, Bangs, Blond_Hair, Bushy_Eyebrows, Chubby, Double_Chin, Eyeglasses, Gray_Hair, No_Beard, Pointy_Nose, Wavy_Hair, Wearing_Hat
Indirectly Influenced Attributes	Oval_Face, Receding_Hairline, Black_Hair, Brown_Hair, Narrow_Eyes

**Table 2:** The comparison of the averaged counterfactual parities for various algorithms on CelebA. The averaged counterfactual disparities are measured for $\lambda_{cf}=0.01,\;0.05$, and 0.1 in the columns cf_reg. The bold number represents the lowest value of averaged counterfactual disparity per attribute.

Attribute	Baseline	cf_aug	cf_reg		
			0.01	0.05	0.1
Arched_Eyebrows	0.202	0.155	0.121	0.086	**0.015**
Attractive	0.122	0.121	0.137	0.084	**0.035**
Bags_Under_Eyes	0.111	0.126	0.024	0.043	**0.008**
Bangs	0.045	0.026	0.028	0.031	**0.005**
Big_Lips	0.116	0.092	0.037	0.043	**0.023**
Big_Nose	0.153	0.116	0.064	0.090	**0.039**
Black_Hair	0.055	0.041	0.011	0.060	**0.004**
Blond_Hair	0.062	0.041	0.036	0.056	**0.010**
Brown_Hair	0.036	0.041	0.072	0.040	**0.024**
Bushy_Eyebrows	0.053	0.055	0.009	0.029	**0.002**
Chubby	0.042	0.028	0.002	0.011	**0.001**
Double_Chin	0.034	0.018	0.002	0.008	**0.000**
Eyeglasses	0.030	0.032	**0.003**	0.018	**0.003**
Gray_Hair	0.016	0.015	0.003	0.016	**0.001**
High_Cheekbones	0.131	0.103	0.195	0.122	**0.021**
Mouth_Slightly_Open	0.106	0.074	0.048	0.053	**0.037**
Narrow_Eyes	0.045	0.024	0.020	0.028	**0.007**
No_Beard	0.194	0.214	0.138	**0.095**	0.134
Oval_Face	0.062	0.053	0.050	0.030	**0.009**
Pale_Skin	0.022	0.024	**0.000**	0.005	**0.000**
Pointy_Nose	0.088	0.096	0.053	0.052	**0.010**
Receding_Hairline	0.036	0.039	0.011	0.015	**0.003**
Smiling	0.108	0.092	**0.060**	0.066	0.064
Straight_Hair	0.034	0.036	0.066	0.055	**0.016**
Wavy_Hair	0.087	0.070	0.087	0.066	**0.029**
Wearing_Earrings	0.121	0.077	**0.009**	0.064	**0.009**
Wearing_Hat	0.029	0.032	0.005	0.014	**0.002**
Wearing_Necklace	0.086	0.076	0.082	0.060	**0.013**
Young	0.144	0.084	0.102	0.066	**0.036**
Average	0.082	0.069	0.0501	0.048	**0.019**

## Data Availability

Not applicable.
